# Adenocarcinoma of the small intestine cohort: prospectively collecting real-world data to improve care and quality of life for patients with a rare cancer

**DOI:** 10.2340/ao.v65.45471

**Published:** 2026-04-28

**Authors:** Pascale J.M. Schafrat, Tim R. de Back, Felice N. van Erning, Frederieke H. van der Baan, Geraldine R. Vink, Miriam Koopman, Ruben S.A. Goedegebuure, Louis Vermeulen, Ignace H.J.T. de Hingh, Dirkje W. Sommeijer

**Affiliations:** a Laboratory of Experimental Oncology and Radiobiology, Cancer Center Amsterdam, Amsterdam UMC and University of Amsterdam, Amsterdam, the Netherlands; b Oncode Institute, Utrecht, the Netherlands; c Amsterdam Gastroenterology Endocrinology Metabolism, Amsterdam, the Netherlands; d Department of Medical Oncology, Amsterdam UMC, Amsterdam, the Netherlands; e Department of Research and Development, Netherlands Comprehensive Cancer Organisation (IKNL), Utrecht, the Netherlands; f Department of Surgery, Catharina Hospital, Eindhoven, the Netherlands; g Department of Medical Oncology, University Medical Center Utrecht, Utrecht University, Utrecht, the Netherlands; h Department of Internal Medicine, Meander Medical Center, Amersfoort, the Netherlands; i Department of Epidemiology, GROW-School for Oncology and Developmental Biology, Maastricht University, Maastricht, the Netherlands; j Department of Internal Medicine, Flevo Hospital, Almere, the Netherlands

**Keywords:** small intestinal adenocarcinoma, prospective, real-world data, quality of life, rare cancer

## Abstract

**Background and purpose:**

Small intestinal adenocarcinoma (SIA) is a rare cancer with a poor prognosis. Optimal treatment strategies are unclear as biological understanding of the disease is limited and randomized controlled trials are lacking. Current management is based on protocols for other gastrointestinal cancers. Therefore, the AdenoCarcinoma of the Small Intestine (ACSI) cohort was initiated, a subcohort of the nationwide Prospective Dutch ColoRectal Cancer cohort (PLCRC) study. The ACSI cohort aims to provide a large-scale, prospective SIA cohort, where clinical and molecular data will be combined to improve knowledge on tumor biology, treatment responsiveness, disease outcome and patient reported outcomes (PROs).

**Patient/material and methods:**

All adult SIA patients in the Netherlands are eligible for inclusion. Nationwide inclusion is facilitated by 68 hospitals. Clinical data are collected, as well as optional PROs, pathology- and blood samples.

**Results:**

Until June 2025, 143 patients have been enrolled. Data of the first 105 patients show a median age of 65 years (interquartile range 58–73) at diagnosis with a slight male predominance (60%). Most tumors are located in the duodenum (57%) and 27% of patients present with stage IV disease, with a majority having single-site metastases (64%). Primary tumor resection is performed in 80%, while systemic treatment is administered in 42%. Four patients did not receive any anti-tumor treatment (14%). Mismatch repair deficiency is detected in 28% of patients.

**Interpretation:**

The ACSI cohort enables structured national collection of clinical data, tumor samples and PROs of SIA patients. These data provide a valuable source for further research and improvement of care for this patient group.

## Introduction

Small intestinal adenocarcinoma (SIA) is a rare and understudied malignancy, with a poor prognosis and a globally rising incidence [1–4]. Rare cancers account for nearly 18% of all solid malignancies; however, recent studies have indicated that survival rates for patients with rare cancers have not improved at the same pace as those for common cancers [5–7]. This lack of progress may be due to limited scientific attention and less commercial funding opportunities for rare cancers such as SIA, resulting in fewer novel treatment options [8]. Consequently, SIA-specific treatment guidelines are scarce and are largely extrapolated from data on more common malignancies, such as colorectal cancer (CRC) [9–11]. There is, however, growing evidence that tumor biology and molecular make-up of SIA differ significantly from other gastrointestinal (GI) cancer types, such as CRC and gastroesophageal cancer [10, 12, 13]. The mechanisms that contribute to the development of SIA remain largely unclear. In addition to the evident knowledge gap regarding tumor biology, important questions on clinical management remain unanswered. For instance, it is unclear whether curative surgery should be combined with (neo-) adjuvant treatment or whether targeted agents with or without chemotherapy have a role in the treatment of SIA [14]. In addition, the impact of the disease on quality of life still needs to be fully elucidated.

Consequently, further research is needed to advance biological understanding, improve the efficacy of treatment and optimize patient reported outcomes (PROs). The first step towards reaching these goals is to prospectively register SIA patients and collect real-world data (RWD). Over recent years, the possibilities, importance and utilization of RWD have been shown with several national prospective databases and cohorts [15–22]. These cohorts succeeded to build comprehensive, nationwide and multidisciplinary research infrastructures. Therefore, the AdenoCarcinoma of the Small Intestine (ACSI) cohort was initiated as a subcohort of the previously established and successfully recruiting Prospective Dutch ColoRectal Cancer Cohort (PLCRC) [18, 23]. The aim of the ACSI cohort is to establish a large-scale prospective SIA cohort, in which clinical and molecular data are integrated to advance knowledge and understanding of tumor biology, treatment responsiveness, disease outcome and PROs. This cohort will provide a comprehensive infrastructure, that combines prospective RWD with tumor specimens, blood samples, and PROs, thereby facilitating research and fostering novel collaborations focused on SIA. Here, we describe the design and preliminary clinical results of the nationwide prospective ACSI cohort study.

## Methods/design

### Design and objectives

The ACSI cohort is launched as a subcohort of PLCRC, following its established infrastructure in accordance with the ‘Strengthening the Reporting of Observational Studies in Epidemiology (STROBE) statement’ guidelines [24]. The addition of the ACSI cohort as a subcohort to the already running PLCRC network was to ensure a fast, efficient and cost-effective study initiation.

### Ethics

The initiation of the ACSI cohort was endorsed by the board of the Dutch Colorectal Cancer Group (DCCG) as the sponsor of PLCRC. As a subcohort of PLCRC, the inclusion of patients with SIA was formalized in the extended approval of PLCRC by the medical ethical review committee of the University Medical Center Utrecht (METC NedMec) in September 2021. PLCRC is registered at Clinicaltrials.gov (NCT02070146), and further documentation is available on www.plcrc.nl. For detailed information regarding the initial approval, see Burbach et al. [18].

### Study population and informed consent

All patients above the age of 18 years, with histologically proven or strong clinical suspicion of SIA (located in the duodenum, jejunum or ileum) are eligible for participation. After identification of eligible patients, the study information and informed consent form (ICF) are provided to the patients. The ICF contains mandatory consent for the collection of longitudinal clinical and survival data. Furthermore, patients may provide optional consent for (1) completing PROs including questionnaires on health-related quality of life, functional outcomes and workability; (2) collection of tumor and other biological material for scientific research; (3) collection of blood samples for scientific research; (4) receiving information regarding studies conducted within the infrastructure of the cohort and (5) being informed about incidental findings. Patients can be enrolled at any stage of the disease and may withdraw their consent unconditionally at any time.

Nationwide enrollment of patients is facilitated by 68 participating centers out of 69 hospital organizations across the Netherlands. After training and the initiation visit of the central PLCRC team, every participating center is responsible for the inclusion of their patients. Included patients are centrally registered.

### Data collection

All clinical data of included patients are routinely collected by trained data managers of the Netherlands Comprehensive Cancer Organisation (IKNL) and registered in the Netherlands Cancer Registry (NCR) according to a SIA-specific item set that was specifically created for this cohort. Collected data focus on patient-, tumor- and treatment characteristics as well as survival. The complete item set with registered variables can be found in Supplementary Table 1. All clinical data are routinely collected 9–12 months after diagnosis and are shared with the study team on a yearly basis.

PROs are routinely gathered by the PLCRC study team. The questionnaires are sent at predefined time points, at the time of signed ICF (baseline), 3, 6 and 12 months after baseline, followed by every 6 months in the second year and yearly from the third year onwards. When distant metastases are diagnosed, questionnaires are sent out every 3 months. Included questionnaires were previously published [18].

With patient consent, all (tumor) tissues, collected as part of diagnostic or treatment procedures, (including, but not limited to, biopsies, resection specimens or liquids) are routinely stored at the local participating hospital before transfer to the regional biobank facilities. Use of tumor tissue for scientific research requires prior approval of the study proposal by the PLCRC scientific board.

In contrast, blood samples are only collected specifically for research purposes and exclusively during routine blood draws associated with specified studies. Prior to the collection of these blood samples for research, approval by the PLCRC scientific board is required.

### Statistics

Standard descriptive statistics were used to assess patient characteristics. Normally distributed continuous variables were reported as mean [standard deviation (SD)], while categorical variables are shown as frequencies with percentages. All statistical analyses were performed using RStudio, version 4.2.2. A *p*-value under 0.05 was considered statistically significant.

## Results

Enrollment of the first patient was in December 2021. Three and a half years after initiation, until June 2025, 143 patients have consented to participate in the ACSI cohort with currently increasing enrollment numbers. Of these 143 patients who agreed with the mandatory consent for the collection of longitudinal clinical and survival data, 139 patients (97%) agreed for the collection of tumor tissue or other biological material, 134 patients (94%) want to be informed about incidental findings, 128 patients (90%) agreed with blood sample collection, 119 patients (83%) gave additional consent for PROs, and 123 patients (86%) agreed to be informed about upcoming trials or studies within the cohort. In the first year, 2022, 71 patients were included, while both in 2023 and 2024, 33 out of the 236 newly diagnosed patients were included in the cohort. This indicates a current coverage of 14% of all newly diagnosed patients [3].

Clinical data from the NCR were shared with the study team in August 2025, which contained the data of the complete registration of the first 105 patients. Patient characteristics are presented in Table 1 and tumor characteristics and therapies in Table 2. Among the included patients, there was a slight predominance of the male sex (60%) with a median age at diagnosis of 65 years (interquartile range [IQR] 58–73). In total, 15 patients (14%) have previously been diagnosed with a predisposing condition. Most tumors were located in the duodenum (57%), followed by the jejunum (25%) and ileum (15%). Stage did not differ between locations of the primary tumor (*p* = 0.227). Mismatch repair (MMR) or microsatellite status was assessed in 78% of patients, with MMR deficiency (dMMR) or microsatellite instability (MSI**-**H) identified in 28% of patients (*n* = 23), irrespective of the primary tumor location. *BRAF* and *RAS* mutational status was largely unknown. Primary resection of the tumor was performed in 84 patients (80%), and most frequently done by a segmental resection (40%) or a pancreatoduodenectomy (36%). Approximately one-third of patients (27%) were diagnosed with stage IV disease (*n* = 28), with the majority having single-organ metastases (64%). In the patients with single-organ metastases, the liver was most affected (29%), followed by the peritoneum (25%) and non-regional lymph nodes (11%). In patients with multi-organ metastases, location of the metastases included combinations of the liver, peritoneum, lymph nodes, lungs, ovaries and spleen. Of all stage IV patients (*n* = 28), 18 patients (64%) received systemic treatment; of which 12 patients (43%) received only palliative systemic therapy and six patients (21%) received palliative surgical resection combined with adjuvant systemic therapy. Six patients (21%) received only palliative surgical resection. Four patients (14%) did not receive any anti-tumor treatment.

**Table 1:** Patient characteristics.

	All patients *n* = 105 (100%)*
**Sex**	
Female	42 (40)
Male	63 (60)
**Age at diagnosis**	
Median [IQR]	65 [58-73]
**Year of diagnosis**	
2016	7 (7)
2017	8 (8)
2018	6 (6)
2019	10 (10)
2020	6 (6)
2021	14 (13)
2022	19 (18)
2023	24 (23)
2024	11 (11)
**Charlson Comorbidity Index (weighted)**	
0	59 (56)
1	30 (29)
2	16 (15)
**Comorbidities**	
Myocardial infarct	5 (5)
Peripheral vascular disease	6 (6)
Cerebrovascular disease	5 (5)
Chronic obstructive pulmonary disease	7 (7)
Diabetes mellitus	11 (11)
with end-organ damage	-
Renal disease	3 (3)
Liver disease (mild/severe)	1 (1)
Ulcer disease	7 (7)
Collagenases	3 (3)
Other malignancy	18 (17)
**Predisposing conditions**	
Celiac disease	4 (4)
Crohn’s disease	4 (4)
Ulcerative colitis	1 (1)
Lynch syndrome	4 (4)
Familial adenomatous polyposis (FAP)	2 (2)

IQR: interquartile range.

* Percentages may not equal 100 due to rounding.

**Table 2:** Tumor characteristics and therapies.

	All patients *n* = 105 (100%)*
**Tumor location**	
Duodenum	60 (57)
Jejunum	26 (25)
Ileum	16 (15)
Not specified	3 (3)
**Grade of differentiation**	
Well	8 (9)
Moderate	56 (64)
Poor	23 (26)
Undifferentiated	1 (1)
*Unknown*	*17*
**Stage at diagnosis**	
I	6 (6)
II	33 (32)
III	37 (36)
IV	28 (27)
*Unknown*	*1*
**Location of synchronous metastases**	
Single-organ metastases	18 (64)
Liver^^^	8 (29)
Peritoneum^^^	7 (25)
Lymph nodes^^^	3 (11)
Multi-organ metastases	10 (36)
**MMR status**	
Proficient	60 (72)
Deficient	23 (28)
*Unknown*	*22*
***BRAF* mutation status**	
*BRAF* wildtype	16 (84)
*BRAF* mutation	3 (16)
*Unknown*	*86*
***RAS* mutated**	
*RAS* wildtype	9 (69)
*KRAS* mutation	2 (15)
Other mutation	2 (15)
*Unknown*	*92*
**Surgical resection**	
No resection	21 (20)
Endoscopic resection	1 (1)
Segmental resection	42 (40)
(Extended) hemicolectomy (right)	3 (3)
Pancreatoduodenectomy	38 (36)
Whipple procedure	11 (11)
Pylorus preserving pancreatoduodenectomy	15 (14)
Pylorus resecting pancreatoduodenectomy	12 (11)
**Systemic therapy**	
None	61 (58)
Neoadjuvant	3 (3)
Adjuvant	28 (27)
Both neoadjuvant & adjuvant	1 (1)
Palliative	12 (11)

MMR: mismatch repair.

* Percentages may not equal 100 due to rounding.

^ Percentages are based on patients with single-organ metastases.

## Discussion and conclusion

Here we present the set-up and first results of a nationwide prospective cohort study combining clinical data, tumor tissue and PROs of patients with SIA. The nationwide and unselected design allows the collection of RWD, facilitates an extensive research platform and provides opportunities for (inter)national collaboration to improve outcomes of patients with this rare disease.

The initial phase of this multi-faceted, nationwide, prospective, observational cohort has been completed, establishing a continuous data source for patients with SIA. Similar efforts for other GI cancers have been made and have proven the feasibility and benefits of prospective nationwide cohorts for improvement of clinical care [17, 18, 23, 25]. Establishing a rare cancer cohort within a 2-year time frame has been achieved through collaboration with and access to the NCR and PLCRC. The existing logistics and network of PLCRC has ensured a fast, efficient and cost-effective start-up. The NCR’s standardized workflow ensures high-quality, nationwide data and enables complete capture, even for patients diagnosed and treated across multiple hospitals. Although the number of included SIA patients is currently still limited, it provides meaningful insights and a valuable foundation for further research. The higher rate of inclusion in 2022 can be explained by the registration of patients diagnosed in previous years who were still under follow-up at the start of the cohort, since patients can be included at any stage of disease. In the following years, the inclusion rate has remained stable. Several factors could explain these small inclusion numbers. First, the integration of a novel cohort into clinical practice is a gradual process, requiring the establishment of routines for the consistent inclusion of patients in the cohort. Second, it can be hypothesized that the rarity of the disease resulted in centralization of care which leads to higher inclusion numbers. However these effects are not yet measurable. Therefore, SIA patients are currently distributed and treated across the country in a widespread manner. With only 200 newly diagnosed SIA patients per year, this equates to roughly three patients per hospital per year. Consequently, the inclusion of all patients remains challenging during the early phases of an established cohort. It is noteworthy that almost all hospitals in the Netherlands, including academic and local hospitals, are affiliated with PLCRC (68 out of 69), thereby making nationwide inclusion of patients in the ACSI cohort possible.

These first results show both similarities and some differences in patient characteristics compared to a previous retrospective Dutch cohort study and the results of the ARCAD-NADEGE cohort study from France [26, 27]. The primary tumor location, stage and location of metastases are comparable across all three studies. The incidences of predisposing conditions, such as celiac disease, Crohn’s disease, or genetic predisposing conditions, like Lynch Syndrome or familial adenomatous polyposis (FAP), are higher in the French cohort, but comparable to previously published nationwide results [28]. Age at diagnosis and sex differences are comparable with the ARCAD-NADEGE cohort [26]. Comparable relatively high incidences of dMMR/MSI-H tumors are found with the Dutch cohort study (28% and 31%, respectively) [27]. Previous literature demonstrated that SIA patients with dMMR/MSI-H most likely benefit from immunotherapy similar to other dMMR/MSI-H solid tumors [29, 30]. However, compared to previous retrospective cohorts, the age at diagnosis is slightly younger in the current results (65 vs 69 and 70 years, respectively) [27, 28] and more frequently affect the male sex (60% vs 52% for both retrospective cohorts, respectively) [27, 28]. A possible explanation for these differences might be related to the retrospective nature of the latter studies, including potential selection bias. Another possibility could be related to the broad eligibility criteria of the ACSI cohort, offering inclusion of patients at any stage in their disease. This might result in an overrepresentation of long-term survivors included during the initial phase of this prospective cohort, reflected in the year of diagnosis. Another potential issue may be that patients with a poor prognosis, a poor performance status or severe symptoms may lack the physical capability or motivation to participate in research during the period of illness and therefore might be underrepresented. Nonetheless, the long-term results of the original PLCRC cohort in 2021 demonstrated comparable results to the Dutch reference population [23]. Therefore, it is hypothesized that these discrepancies will diminish as time progresses.

The current limitations of the cohort, including potential areas of unmet need, are acknowledged. First, the absence of molecular marker assessment in a large proportion of patients is likely attributable to their limited impact on current clinical decision-making. Previous studies have already shown that *BRAF* mutations in SIA are usually non-V600E variants, in contrast to CRC [12, 31]. The current collection of tumor tissue available within the cohort offers opportunities to additionally gather these molecular data for research purposes and to provide recommendations for standardization in clinical practice. Furthermore, due to short follow-up, survival data are not yet reliable. These shortcomings of the cohort are expected to decrease with higher inclusion numbers and longer available follow-up data. Second, the improvement of inclusion numbers by incorporating patient enrollment into clinical routine should remain a focus point but is expected to further improve over time. The current limitations underscore the necessity for enhanced recognition and comprehension of rare cancers such as SIA. Following the establishment of prospective registration cohorts, future research could be expanded by clinical trials and eventually towards improved disease knowledge and improvement of care. Future analyses of the cohort, together with currently ongoing clinical trials, such as the BALLAD trial (a phase III trial to assess post-operative chemotherapy in localized SIA) and the PRODIGE 86 trial (a phase II trial to evaluate mFOLFIRINOX and mFOLFOX in the treatment of locally advanced or metastatic SIA) would further aid the interpretation of the results and help assess the generalizability of both the findings from these trials, as well as our findings from the ACSI cohort [32, 33].

In summary, the ACSI cohort enables structured collection of RWD, tumor tissues, blood samples and PROs for SIA patients, on a nationwide level. This growing dataset provides a valuable source for further research initiatives and improvement of care for this patient group with a rare cancer. For example, the ACSI cohort can serve multiple purposes, such as acting as a real-world control arm for single-arm phase 2 trials, enabling longitudinal monitoring of treatment outcomes and PROs, and support the availability of tissue to enhance biomarker- or translational research.

## Supplementary Material



## Data Availability

The coded data that support the findings of this study are available from the corresponding author upon reasonable request.

## ACSI Study Group

P.J.M. Schafrat, D.W. Sommeijer, I.H.J.T. de Hingh, R.S.A. Goedegebuure, L. Vermeulen, T.R. de Back, M. Chalabi, E. Gootjes, L. Mekenkamp, E. Dekker, A. Farina Sarasqueta, G. Kazemier, M.G.H. Besselink, M. Koopman, G.R. Vink, L. Simkens, J.B. Tuynman, L. van Iersel, F. Mols, J.W. Wilmink, H.W.M. van Laarhoven, T.E. Buffart, N.C.T. van Grieken, M.L. Wumkes, L.E. Nijman, F.N. van Erning, S.A.W. Bouwense, B. Mostert, R. Fijneman, Stichting Darmkanker.

## PLCRC Study Group

M. Los, J.M. van Dodewaard, M.L.R. Tjin-A-Ton, A.M.T. van der Velden, A.H.W. Schiphorst, M. Koopman, K. Talsma, B.M.J. Scholtes, D.W. Sommeijer, H.B.A.C. Stockmann, D.D.E. Zimmerman, P.J. Tanis, M. Vermaas, W.J. Vles, M. Verseveld, M. Tent, J.W.B. de Groot, L.B.J. Valkenburg, K.C.M.J. Peeters, J.H.W. de Wilt, R. Blankburgh, N.F.M. Kok, P.P.L.O Coene, A.U.G. van Lent, A. Goossens, E.J.Th. Belt, J.W.T. Dekker, I.H.J.T. de Hingh, M. Berbée, R.W.M. Schrauwen, F.J.F. Jeurissen, R. Hoekstra, M.B. Polée, D. Houtsma, M.J. Mekenkamp, M.P. Hendriks, M.R. Meijerink, H. van Cruijsen, J. Schreinemakers, J. Jansen, J. Konsten, P. Nieboer, N.A.J.B. Peters, M.P.S. Sie, T. van Voorthuizen, F. Terheggen, G.H.E.J. Vijgen, G.A.P. Hospers, J.F.M. Pruijt, L. Simkens, A.I. de Vos, D.J.A. Sonneveld, M. Cloos-van Balen, A.P. Schouten van der Velden, L.E.A. Spierings, A.W. Haringhuizen, J. Willems, R.P. Veenstra, J.A. Wegdam, M.A. Davidis, J. Janssen, P. van Duijvendijk, T. Kuiper, R. van de Wetering, M. Blussé-van Oud Alblas, J. de Boer, F. de Boer, L. Steensel
