# Supplementary material for: Adenocarcinoma of the small intestine cohort: prospectively collecting real-world data to improve care and quality of life for patients with a rare cancer
Source: Acta Oncol. 2026 Apr 28;65:45471. doi: 10.2340/ao.v65.45471 (PMC13130176; doi:10.2340/ao.v65.45471)
Supplement: Supplementary file 1 [file AO-65-45471-s1.pdf]

Supplementary material has been published as submitted. It has not been copyedited, or typeset by Acta Oncologica

Supplementary table 1. Item set of collected data of patients with small intestinal adenocarcinoma.

Patient characteristics

- Sex
- Age at diagnosis
- Year of diagnosis
- Vital status
- WHO performance status before start therapy
- Charlson Comorbidity Index
- Additional comorbidities
  - Celiac disease
  - Crohn’s disease
  - Ulcerative Colitis
  - Lynch Syndrome
  - Familial Adenomatous Polyposis (FAP)

Tumor characteristics

- Tumor location
- Base of diagnosis
- Grade of differentiation
- Stage (clinical and pathological)
- Lymphnode ratio
- Angio invasion (in case of T1 tumors)
- Tumor budding (in case of T1 tumors)
- Location of metastases
- Number of tumor deposits (if possible)
- Mismatch repair (MMR) status
- RAS mutation status
- BRAF mutation status
- Her2Neu status and type

Surgical characteristics

- Surgical technique in all surgical treatments
- Urgency in all surgical treatments
- Radicality of resection
- Gastroenterostomy
- Endoprothesis/stent
- Bypass
  - Type of bypass
  - Route of anastomosis
  - As treatment
  - Surgical technique of bypass
- Complications of surgery
  - Surgical site infection
  - Leakage

Treatment characteristics

- Administration of systemic treatment
- Type of systemic or targeted treatment
  - 5-FU
  - Capecitabine
  - Oxaliplatin
  - Bevacizumab
  - Trastuzumab
  - Pertuzumab
  - Pembrolizumab
  - Nivolumab
- Number of cycles of systemic and targeted therapy
  - Response on therapy (based on imaging assessment of the radiologist and if available pathological response according to the Mandard classification)

Follow-up after 3 years

- Recurrence / progression (including location of distant metastases, interval data)
- All received treatments
- Interval until last follow-up
